# The Significance of Adaptation and Coping with Disease among Patients with Diagnosed Gynaecological Cancer in the Context of Disease Acceptance

**DOI:** 10.3390/ijerph19127218

**Published:** 2022-06-13

**Authors:** Sylwia Wieder-Huszla, Joanna Owsianowska, Anita Chudecka-Głaz, Dorota Branecka-Woźniak, Anna Jurczak

**Affiliations:** 1 Department of Clinical Nursing, Pomeranian Medical University in Szczecin, 71-210 Szczecin, Poland; joanna.owsianowska@pum.edu.pl (J.O.); anna.jurczak@pum.edu.pl (A.J.); 2 Department of Gynaecological Surgery and Gynaecological Oncology of Adults and Adolescents, Pomeranian Medical University in Szczecin, 70-111 Szczecin, Poland; anita.chudecka@pum.edu.pl; 3 Department of Gynaecology and Reproductive Health, Pomeranian Medical University in Szczecin, 71-210 Szczecin, Poland; dorota.branecka@pum.edu.pl

**Keywords:** women, gynaecological cancer, disease acceptance, mental adaptation, adaptation

## Abstract

Uterine/endometrial and ovarian tumours are among the most common gynaecological cancers. Adaptation to cancer encompasses a variety of complex behavioural, cognitive, and emotional processes. The purpose of mental adaptation is to alleviate emotional discomfort and regain mental stability. The aim of the study was to assess the influence of adaptation and coping with gynaecological cancer on the level of disease acceptance among the studied women. The study included 81 patients diagnosed with gynaecological cancer. Mental adaptation to cancer was measured using the Min-Mac scale, disease acceptance was measured using the AIS and the level of adaptation was measured using the CAPS. The average AIS score was 26.65 ± 8.85 points. Adaptation and coping methods did not vary significantly depending on the diagnosed type of cancer. The constructive style of fighting the disease prevailed (45.11 ± 6.01). The AIS scores correlated significantly and positively with the intensity of the constructive style of mental adaptation, and negatively with the intensity of the destructive style. The studied group of patients with gynaecological cancer displayed a moderate level of disease acceptance, the constructive style of adaptation was the most prevalent, and the location of the cancer did not have an effect on coping mechanisms.

## 1. Introduction

Cancers are a complex health and social issue. It is estimated that, in 2020, 18 million new cancer cases were diagnosed, 8.8 million of which were found among women. Equally disturbing is the estimated mortality rate of around 500,000 deaths annually [1,2]. Uterine body cancer, endometrial cancer, and ovarian cancer are among the most frequently diagnosed gynaecological cancers. Each of these types of cancer presents different epidemiologic characteristics, genetic risk factors, symptoms, prognoses, and responses to the administered treatment [2,3].

Uterine body cancer/endometrial cancer is the most frequently occurring cancer among women. The available epidemiologic data confirms that there is a growing number of cases in developed countries, including Poland. According to data published by the Polish National Cancer Registry, over 5000 endometrial cancer cases are diagnosed in Poland annually [4,5]. In the United States, it is the fourth most common malignant cancer among women. The risk factors include: the endometrium’s high exposure to oestrogens, early first menstruation, late menopause, tamoxifen therapy, infertility or lack of ovulation, and polycystic ovary syndrome. A significant risk factor is age, as the vast majority of cases are found among women over 50, and the highest number of cases is found among women between 65 and 69. Other possible determinants are obesity, arterial hypertension, diabetes, and genetic factors. The fundamental treatment method is hysterectomy with bilateral salpingo-oophorectomy, but radio- and chemotherapy also play an important role. The five-year survival rate is around 77%, but survival time strongly depends on both the stage of the disease, and on the histological type of the cancer [6,7].

Ovarian cancer is the fifth most common cause of women’s death, and the most fatal of all gynaecological cancers. Over 220,000 new cases are diagnosed globally each year, and around 150,000 women die as a result of this cancer. Around 14,000 new cases are found annually in the United States alone [8,9]. In Poland, over 3500 new ovarian cancer cases are diagnosed each year [5]. This type of cancer can derive from epithelial, embryonic, and stromal cells. Epithelial ovarian cancer is the most frequent, and the most fatal of the variants. The histological types of epithelial ovarian cancer include: high-grade and low-grade serous carcinomas, endometrial cancer, clear cell cancer, and mucinous carcinoma [10]. Currently, it is understood that most serous carcinomas stem from the fallopian tube, but the umbrella term of ovarian cancers is still applied to them [11]. In light of this knowledge, the cytological subtypes of high-grade and low-grade malignancy become a more relevant distinction, since serous ovarian carcinomas of low-grade malignancy may develop more slowly, but are also more resistant to chemotherapy than serous ovarian carcinomas of high-grade malignancy [10]. First-line therapy given for ovarian cancer has evolved in recent decades from administering a single alkylating agent to the current standard of cytoreductive surgery, followed by systemic treatment based on administering anticancer drugs. First-line chemotherapy involves a dosing regimen of platinum derivatives and paclitaxel administered in six courses in 21-day intervals [12,13,14,15].

Early stages of cancer rarely result in observable symptoms. They usually occur only when the cancer infiltrates neighbouring organs, which means that the disease is advanced [16]. Adaptation to cancer encompasses many behavioural, cognitive, and mental processes. Mental adaptation is usually considered synonymous with stress management when undergoing traumatic experiences. Adaptation alleviates emotional discomfort and allows one to regain mental stability. Its main purpose is coping with the disease, treatment methods, and significant changes that occur in one’s life due to the disease [17]. The concept of adaptation to cancer is derived from Lazarus and Folkman’s theory. These authors defined coping as “constantly changing cognitive and behavioural efforts to manage specific external and internal demands that are appraised as taxing or exceeding the resources of the person” [18]. Thus, the main assumption of this theory is that stress is a particular relationship between the individual and the environment. For a person it is not only the event itself that matters, but also how it is perceived and interpreted. Therefore, actions are motivated both by individual differences stemming from a person’s character, and by the significance people ascribe to the external situation [18,19]. Lazarus and Folkman distinguish two functional coping strategies: instrumental and regulatory. The instrumental strategy is focused on solving the problem, whereas the regulatory strategy aims at controlling the emotional response evoked by a particular stimulus. The body’s action to deal with the threatening problem is described in the literature as a process, style, or strategy. A coping strategy is, then, an individual’s reaction to the situation [20].

Furthermore, Parker and Endler have distinguished three stress-coping styles: task-oriented coping, emotion-oriented coping, and avoidance-oriented coping. A task-oriented person undertakes actions in order to solve the problem or attempts to change the situation. An emotion-oriented person focuses on regulating their feelings and emotional response to the problem instead of addressing it. Avoidance-oriented coping is characterized by denying, minimizing, or otherwise avoiding dealing directly with a stressful situation [21]. Regarding oncological patients, two coping styles can be distinguished based on their psychological characteristics: the constructive style and the destructive style. The constructive style involves a fighting spirit and positive re-evaluation. The destructive style, conversely, is comprised of anxiety preoccupation and helplessness–hopelessness, which results in feeling worthless, lost, and passively giving in to the disease. A fighting spirit allows one to treat the disease like a personal challenge, and, consequently, to undertake actions to overcome it [16]. While the literature provides numerous studies on the acceptance of the disease, little attention is paid to adaptation to cancer and coping with it. Therefore, it is necessary to enrich the knowledge of adaptation processes. Coping strategies and disease acceptance significantly affect the treatment process in many diseases, including cancer. Patients with a high level of acceptance of the disease cope better with pain in cancer and choose active coping strategies, which translates into therapeutic effects [22,23,24]. The available studies seem to confirm that cancer patients tend to choose constructive strategies of coping with the disease [7,22,23,25,26,27], which, according to Rogala et al., may determine mental adaptation to the disease [16]. The aim of this study was to assess the influence of adaptation to and coping with gynaecological cancer on the level of disease acceptance among the studied women.

## 2. Materials and Methods

### 2.1. Study Design

This survey-based study involved a group of 81 female patients undergoing treatment in the Clinic of Gynaecological Surgery and Gynaecological Oncology of Adults and Adolescents, the Pomeranian Medical University in Szczecin. A necessary requirement to take part in the study was giving informed consent. The study was conducted in accordance with the Declaration of Helsinki, and the protocol was approved by the Bioethics Committee (Resolution no. KB-0012/81/18). All patients included in the study had been diagnosed with advanced ovarian cancer or uterine body/endometrial cancer, and underwent surgical treatment followed by chemotherapy.

### 2.2. Research Instruments

This survey-based study was performed using the following standardised research instruments:

- Mental Adaptation to Cancer Scale (Mini-Mac) by M. Watson et al., as adapted by Z. Juczyński [25];
- Acceptance of Illness Scale (AIS) by B.J. Felton et al., as adapted by Z. Juczyński [25];
- Coping and Adaptation Processing Scale (CAPS) by C. Roy [28].

Additionally, an original questionnaire was used to obtain basic sociodemographic data (age, place of residence, employment status, education, marital status), medical data (menstruation, history of cancer in family, medication administered), and information on physical activity.

#### 2.2.1. Mental Adaptation to Cancer Scale (Mini-Mac)

The Mini-Mac scale allows researchers to evaluate how a person adapts to cancer, and how they cope with the disease and its symptoms, such as pain, fatigue, and poor wellbeing. It contains 29 statements, which are used to assess four domains—coping strategies divided into constructive (fighting spirit and positive re-evaluation) and destructive (anxiety preoccupation and helplessness-hopelessness) strategies. Each statement is evaluated on a four-point scale: from 1—definitely not to 4—definitely yes. The points are summed for each strategy separately. The final scores may range from 7 to 28 points. Then, the results are converted into sten scores, which are interpreted as follows: 1–4 sten (10–24 points)―low; 5–6 sten (25–29 points)―average; and 7–10 sten (30–40 points)―high. The higher the score, the lower the intensity of behaviours characteristic of a particular coping strategy [25].

#### 2.2.2. Acceptance of Illness Scale (AIS)

The AIS is used to evaluate the degree to which the patient accepts their condition. It is comprised of eight statements describing the negative consequences of ill health. The subject evaluates their current state in relation to each of the sentences on a five-point scale: from 1—strongly agree to 5—strongly disagree. The general result of the disease acceptance measurement is the sum of points that may range from 8 to 40. The final results are divided into three ranges: a score below 19 points reflects weak acceptance, a score between 20 and 35 points—moderate acceptance, and a score above 36—full acceptance of and adaptation to the disease [25].

#### 2.2.3. Coping and Adaptation Processing Scale (CAPS)

The CAPS is used to evaluate how a person reacts to crisis and difficult life situations, such as illness, or a recent traumatic event. The questionnaire is comprised of 47 statements, which are evaluated on a four-point scale: from 1—never to 4—always. The subject may score between 47 and 188 points. The CAPS allows insight into the general adaptive and coping capabilities of the subject. It enables researchers to analyse the functioning of a person in five dimensions: interdependence, physiological dimension, self-concept, roles in society, and coping. The higher the score obtained by the patient, the better they cope and adapt [28].

### 2.3. Statistical Analysis

Statistical analysis was conducted using MedCalc version 20.014 (Ostend, Belgium). Normal distribution of the continuous variables was verified using Shapiro—Wilk’s test. Since the distribution of most of the continuous variables did not deviate significantly from normal, means and standard deviation were used in their description. The qualitative variables are presented as numbers and percentages. Consequently, statistical testing was based on parametric tests, namely Student’s *t*-test and analysis of variance (ANOVA), depending on the number of independent variables. Post-hoc analyses were performed using Tukey’s test, and the continuous variables were correlated using Pearson’s method. Co-occurrence of qualitative variables was calculated using the chi^2^ test and Fisher’s exact test. Predictors of disease acceptance were chosen using logistic regression. A two-tailed significance level of *p* = 0.05 was adopted. MedCalc^®^ Statistical Software version 20.106 (MedCalc Software Ltd., Ostend, Belgium; https://www.medcalc.org (accessed on 1 January 2022)) was used for calculations.

## 3. Results

### 3.1. Characteristics of the Study Sample

The study included 81 women diagnosed with uterine cancer (n = 58, 71.6%) or ovarian cancer (n = 23, 28.4%). The average age of the studied women was 60.31 ± 11.72 years (Me 62 years, IQR: 54–68.5; min. 34 years, max. 85 years). The analysis revealed a statistically significant relationship between the age of the subjects and the occurrence of ovarian cancer. The women who suffered from ovarian cancer were significantly younger compared to their counterparts with uterine cancer (54.95 ± 12.29 vs. 62.43 ± 10.89; *p* = 0.009). None of the sociodemographic variables were significantly related to cancer type (Table 1).

### 3.2. Adaptation to Cancer Scale (Mini-Mac)

Analysis of the Mini-Mac scale results did not reveal any significant differences in the scores for any of the subscales depending on the type of cancer (Table 2).

**Figure 1:**
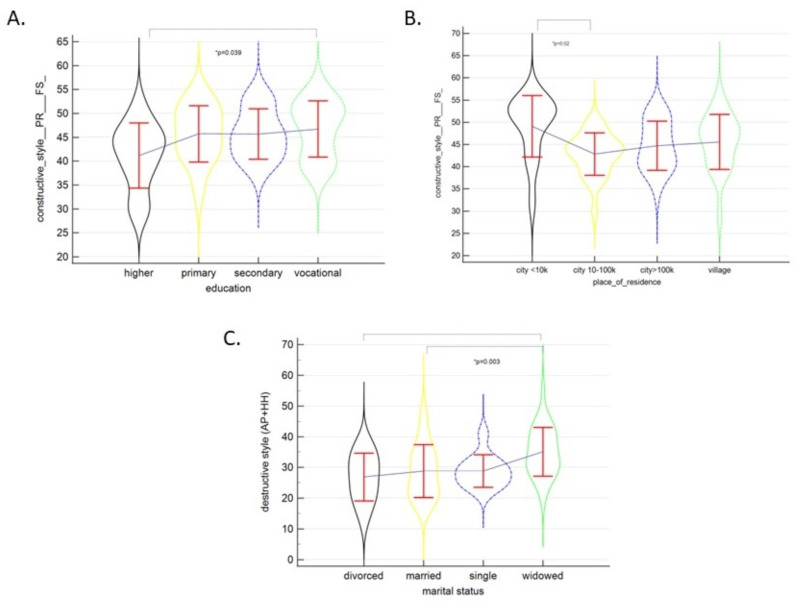
The constructive style by education (**A**) and place of residence (**B**), and destructive style by education (**C**). Means and standard deviations are given. * *p* < 0.05.

The women with vocational education scored significantly higher for the constructive style domain than their counterparts with higher education (*p* = 0.039) (Figure 1A). Similarly, those who lived in smaller cities (≤10,000 inhabitants) scored higher for this domain than those who lived in large cities of over 100,000 inhabitants (*p* = 0.02) (Figure 1B). No differences were observed with regard to marital status (*p* = 0.48) and employment status (*p* = 0.91) for this subscale. The intensity of the destructive style was not dependent on education (*p* = 0.68), place of residence (*p* = 0.36), or employment status (*p* = 0.46). However, those who were divorced or married scored lower on this subscale than widows (*p* = 0.003) (Figure 1C).

The destructive style score significantly increased with age in the group of women with uterine cancer. This statistical tendency was observed for the whole study sample (Table 3, Figure 1).

Converting the results into sten scores, and then into the categories of mental adaptation, did not reveal any statistically significant relationships between constructive and destructive styles and the diagnosed type of cancer (Table 4).

**Figure 2:**
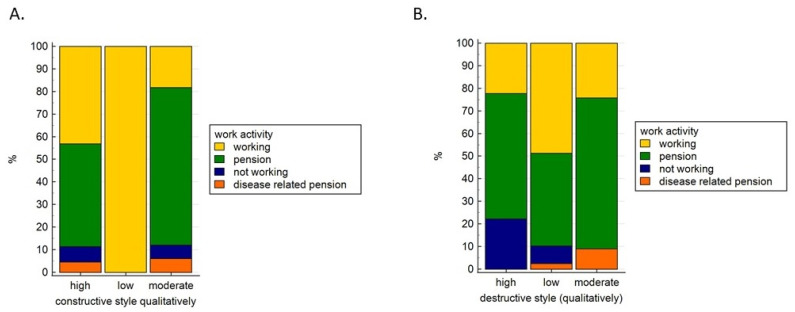
Qualitative mental adaptation styles constructive (**A**) and destructive (**B**) regarding present work activity constructive.

Analysis of the qualitative categories of mental adaptation with regard to sociodemographic variables demonstrated that both constructive and destructive styles depend on employment status (Figure 2).

### 3.3. Acceptance of Illness Scale (AIS)

The patients obtained an average score of 26.65 ± 8.85 points on the AIS. The participants presented a similar (*p* = 0.47) level of acceptance, which is evidenced by the number of points scored on the AIS by the ovarian cancer patients (27.78 ± 8.93), and by the uterine cancer patients (26.21 ± 8.86). None of the sociodemographic variables were significantly related to the level of disease acceptance (Table 5). The AIS scores were not significantly correlated with the age of the women (entire group: r = −0.113, *p* = 0.32; ovarian cancer patients—r = −0.02, *p* = 0.93; uterine cancer patients: r = −0.126, *p* = 0.34).

### 3.4. Coping and Adaptation Processing Scale (CAPS)

**Figure 3:**
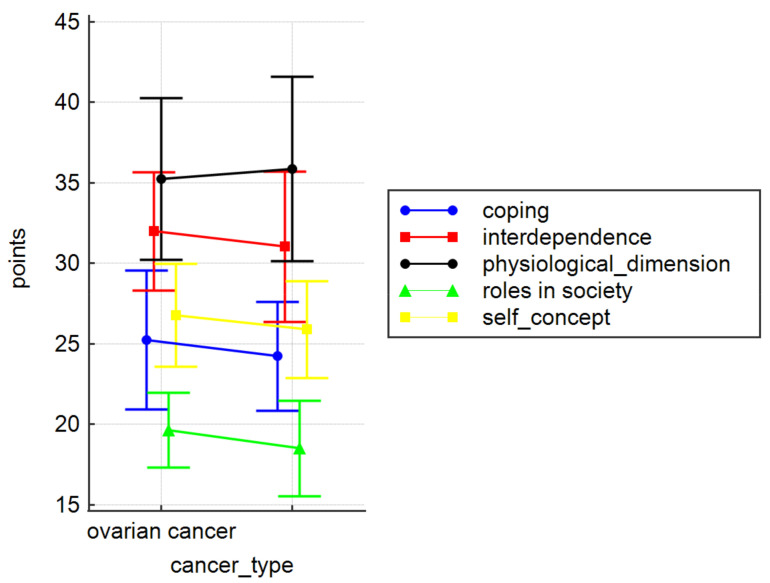
CAPS subscales by diagnosis. Means and standards deviations are given.

In our study, adaptation and coping did not vary significantly depending on the diagnosis, for any of the CAPS subscales (coping: *p* = 0.26; interdependence: *p* = 0.38; physiological dimension: *p* = 0.66; roles in society: *p* = 0.10; self-concept: *p* = 0.24) (Figure 3) or the total CAPS score (ovarian cancer: 138.95 ± 14.99; uterine cancer: 135.57 ± 14.61; entire group: 136.53 ± 14.70).

**Figure 4:**
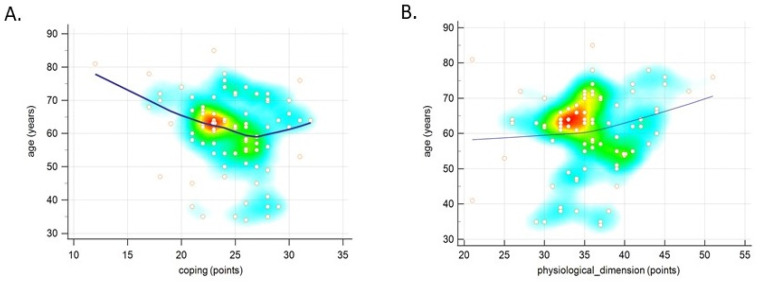
Correlation of age with CAPS subscales: coping (**A**) and physiological dimension (**B**) in all study participants.

Having analysed which of the sociodemographic data is strongly tied to the intensity of the adaptive qualities described by the CAPS (Table 6), it was noticed that the patients who lived in large cities (≤100,000 inhabitants) scored significantly lower on the physiological dimension subscale compared to those who lived in smaller cities (fewer than 10,000 inhabitants). Additionally, it was found that the number of points on this subscale correlated strongly and positively with the age of the women diagnosed with uterine cancer (r = 0.3, *p* = 0.02). In the entire study sample, a tendency for negative correlation between age and the coping subscale scores (r = −0.22, *p* = 0.05), and for positive correlation between age and the physiological dimension subscale scores (r = 0.21, *p* = 0.06), was found. The results are shown in Figure 4.

### 3.5. Acceptance of the Disease and Adaptation to Cancer, and Mental Adaptation

**Figure 5:**
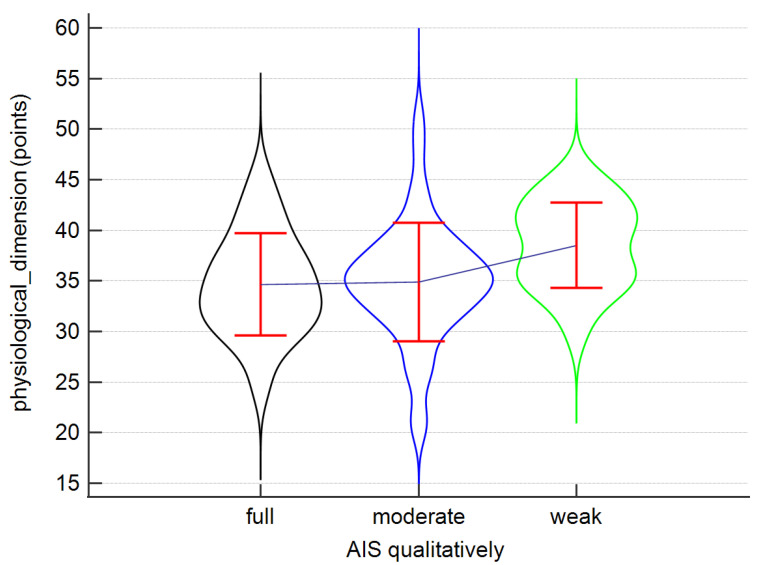
Number of points for the CAPS physiological dimension domain and the level of disease acceptance. Means and standard deviations are given.

To assess whether the psychological variables are somehow related, scores for all three mental subscales were correlated. The AIS score was found to correlate moderately and negatively with the CAPS score in the physiological dimension domain, both in the entire study sample and in the group with uterine cancer. Similarly, those with poor acceptance of the disease scored higher in the physiological dimension domain compared to those with a moderate level of acceptance of the disease (*p* = 0.03) (Table 7, Figure 5).

**Figure 6:**
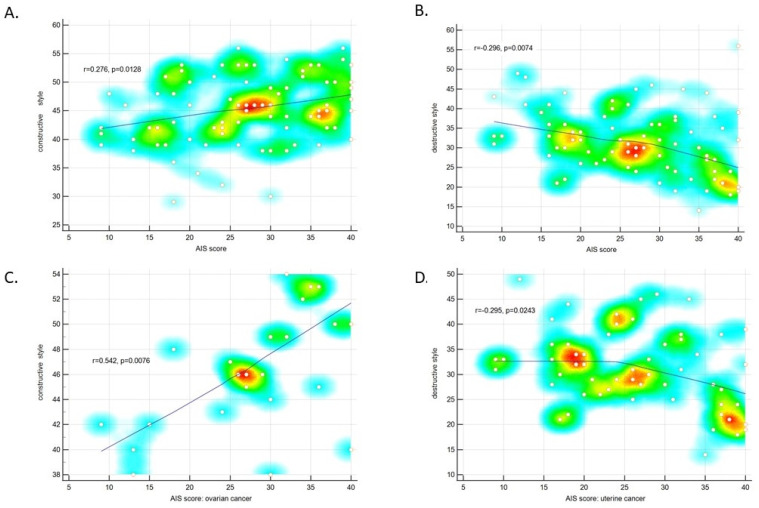
Correlation between the AIS scores and the intensity of mental adaptation styles: the constructive mental adaptation style (**A**), the destructive mental adaptation style (**B**), the constructive style among women with ovarian cancer (**C**), the destructive style among women with uterine cancer (**D**).

The AIS scores also correlated significantly and positively with the intensity of the constructive mental adaptation style, and negatively with the intensity of the destructive mental adaptation style. These correlations were found in the entire study sample. Regarding the division into the two cancer types, we observed the positive correlation between the AIS scores and the constructive style among women with ovarian cancer, and the negative correlation between the AIS scores and the destructive style among women with uterine cancer (Figure 6).

The subjects who fully accepted the disease reached significantly higher scores on the constructive adaptation subscale compared to those who adapted poorly (full acceptance: 47.22 ± 4.95; moderate acceptance: 45.45 ± 6.28; poor acceptance: 42.31 ± 5.47; *p* = 0.037). Regarding the destructive style, those who fully accepted the disease obtained the lowest scores (full acceptance: 28.5 ± 10.37; moderate acceptance: 31.48 ± 7.14; poor acceptance: 34.68 ± 8.45; *p* = 0.08). However, when both of the variables were expressed qualitatively, no significant dependencies were found between the level of disease acceptance and mental adaptation.

## 4. Discussion

Analysis of the subject literature indicates that a patient’s appropriate response to the disease improves therapeutic effects. According to many studies, coping mechanisms, disease acceptance, and adaptation to living with the disease are concepts that have a tremendous influence on the cancer treatment process [25,29]. It has indeed been observed that patients who display a high level of disease acceptance cope with cancer-related pain better, and choose more effective coping strategies, which has a direct effect on the therapeutic effect [30].

The complexity of the female reproductive system predisposes to the development of many squamous cell carcinomas that have a wide variety of features. Adopting a specific attitude towards the disease (constructive or destructive adaptation) affects not only the patient’s quality of life, but also the long-term effects of treatment. Looking at the issue from this perspective, it becomes clear that assessment of coping strategies is useful at all stages of treatment and convalescence [16,30].

When the patient adopts a fighting spirit and resists the disease, it may increase the chances of survival, as confirmed by studies conducted by Malicka et al. on women who had undergone mastectomy [31]. According to these authors, both stoic acceptance of the disease, and feeling hopeless and helpless, may make it more difficult to mobilise oneself to fight the disease. It may also interfere with defence mechanisms, thus lowering the survival rate. At the same time, the researchers assert that strategies such as denial, minimising, and avoidance may be valuable in the early stages of cancer battle, but are problematic when they become the primary coping mechanisms, as they may lead to escaping the problem [31]. Rogala et al. used the Mini-Mac test to analyse mental adaptation to disease in a group of 30 women who had undergone surgical treatment for cervical cancer. The subjects reached the highest scores for the fighting spirit (22.63) and positive re-evaluation (21.10) domains. The lowest scores were noted for helplessness and hopelessness (12.63), showing that the constructive strategies were more prevalent [32]. Analysis of the sociodemographic data revealed that the women who were married or in relationships scored higher for helplessness and hopelessness and for positive re-evaluation compared to the patients who were single. The authors also observed that the patients who claimed to be in a bad or moderate financial situation showed a stronger tendency to rely on destructive strategies. Other variables, including education, had no effect on the choice of a strategy [32]. In our study, the constructive strategies of mental adaptation were also more frequently adopted. The highest scores for the constructive domains were obtained by the patients who had vocational education and lived in small cities. Destructive behaviours, on the other hand, were mostly displayed by widows. The number of points scored for the destructive domains also increased significantly with the age of the subjects, especially among those diagnosed with uterine cancer. Kupcewicz et al. conducted a study of 102 women with histopathologically confirmed gynaecological cancer, among which endometrial cancer was the most common (94.1%) [33]. In our study the group of subjects was also comprised largely of patients with uterine cancer (71.6%). The average age of the participants in Kupcewicz et al.’s study [33] was 56.10 years (SD = 10.75), which is similar to the average age of the participants in our study (M = 60.31; SD = 11.72). Kupcewicz et al.’s study [33] revealed that the scores for the constructive style domain (43.5 ± 5.76) were significantly higher than those for the destructive style (21.7 ± 5.28). This means that the studied women chose active approaches to the disease, such as a fighting spirit (M = 21.6 ± 3.47) and positive re-evaluation (M = 21.9 ± 3.01) [33]. Analogous results were found in our study, in which patients scored higher for the constructive domains (M = 45.11 ± 6.01) than for the destructive domains (M = 31.57 ± 8.41) regardless of the type of cancer. This was manifested as perceiving the disease as a challenge and taking action against it. A similar observation was made by Religioni et al. [7], who analysed patients with endometrial cancer, receiving outpatient care after completion of oncological treatment. They also reported that positive re-evaluation was statistically significantly related to age and employment status; this strategy was more common among older women and pensioners. Conflicting results were noted by Kupcewicz et al. According to these authors, it was the older patients who reached lower scores for fighting spirit, and higher scores for helplessness and hopelessness [33,34]. As stated by Religioni et al., patients with ovarian cancer displayed active adaptation. At the same time, they showed that the level of acceptance depended on the economic situation―a lower income per member of the patient’s household was associated with an increase in the score for helplessness and hopelessness [35]. This thesis is supported by the results of our study, in which women with a regular source of income, regardless of its form (work or pension), adopted a constructive style and an active attitude towards the disease.

Understanding the mechanism of disease acceptance and, above all, the way it is conditioned, plays an important role in chronic diseases, including cancer. According to Juczyński, the AIS can be a good predictor of disease-related quality of life, which is synonymous with life satisfaction and current assessment of health [25].

The assessment of disease acceptance using the AIS conducted by Religioni et al. in women with gynaecological cancers showed a score of M = 27.08 (SD = 7.48) for patients with endometrial cancer [7] and M = 26.73 (SD = 7.28) for patients with ovarian cancer [35]. The analysis carried out by the authors did not reveal any statistically significant relationships between the sociodemographic variables and the level of disease acceptance. Better adaptation to the disease was indicated only by higher scores of the AIS in ovarian cancer patients who had a higher income and were not undergoing chemotherapy. In our study, the mean AIS test score was similar at 26.65 points (SD = 8.85), including among patients diagnosed with ovarian cancer (27.78 ± 8.93) and uterine cancer (26.21 ± 8.86). Better adaptation to the disease was only indicated by higher results of the AIS obtained by patients with ovarian cancer who had a higher income and were not undergoing chemotherapy. In our study, the mean AIS score was similar and amounted to 26.65 points (SD = 8.85), including patients diagnosed with ovarian cancer (27.78 ± 8.93) and uterine cancer (26.21 ± 8.86). The level of disease acceptance did not depend on sociodemographic factors. The results of Czerw et al.’s study, which assessed the acceptance of the disease among breast cancer patients whose mean AIS score was 28.45 ± 7.98, showed that the differentiating factors were net income per household member (*p* = 0.01) and place of residence (*p* = 0.035). Women with a higher income and those living in large cities scored higher, which means that they adapted better to the disease [22]. In Cipora et al.’s study [36], conducted among women with breast cancer, the mean test result was 26.53 ± 7.71, with more than half of the participants (50.6%) having an AIS score between 20 and 29 points. Of the variables adopted by the researchers, age and employment status were those that significantly differentiated the AIS score. In the study by Czerw et al., the highest mean score (23.43) was obtained for the fighting spirit subscale, but the mean scores were not significantly differentiated by socioeconomic variables [22]. Analysis of our results showed that the AIS scores correlated with a greater intensity of the constructive style among the studied patients. Women who fully accepted their disease reached significantly higher scores on the subscale of constructive adaptation compared to those with poor disease acceptance. In our study, patients with ovarian cancer were characterized by better adaptation and a more active approach to coping with the disease. A further study of patients with breast, ovarian, endometrial, or colorectal cancer, conducted by Czerw et al. [37] using the Mini-Mac questionnaire, revealed that the fighting spirit and positive re-evaluation strategies―characteristic of the constructive coping style—were the most prevalent among the subjects. The adoption of such an approach proves that the patient’s strong coping mechanisms have been activated, which contributes to a better prognosis and better long-term therapeutic effects [31]. Our study, in which the examined patients were most often characterized by constructive styles, confirms this key aspect of the results obtained by Czerw et al. [37].

Acceptance of the disease at a good level and well-developed adaptive mechanisms allow the patient to feel better not only physically, but also mentally. This, in turn, allows them to gain more confidence in the therapeutic team and accept the proposed treatment regimens [25]. Currently available studies are focused more on the level of acceptance and mental adaptation to the disease, without delving into the particular adaptive mechanisms.

In our study, we attempted to assess the adaptive capabilities of cancer patients based on the CAPS. This tool allows insight into the general adaptive and coping capabilities of an individual who has faced the difficult life situation of being diagnosed with cancer. The analysis showed that the mean score obtained by ovarian cancer patients was 138.95 ± 14.99 points, and the mean result of uterine cancer patients was 135.57 ± 14.61 points, with a possible score ranging from 47 to 188 points. However, it was not confirmed whether coping mechanisms of the patients differed significantly by diagnosis for any of the subscales (coping, interdependence, physiological dimension, roles in society, self-concept). Sociodemographic variables were closely linked to the level of adaptive skills, as patients who lived in large cities scored significantly lower on the physiological dimension subscale. However, it was observed that the age of patients with uterine cancer correlated with the number of points on this subscale. Regardless of the type of cancer, the older the woman, the greater the deficit in the coping subscale, and the smaller the deficit in the physiological dimension subscale. The preliminary results of the study conducted by de Groot et al. show that the marital status of women suffering from gynaecological cancer has an effect on their psychosocial state. Relationships with their partners and significant others may improve their functioning, but may also exacerbate symptoms and anxiety related to illness and treatment. An individualised approach to this group of patients can make a difference to their quality of life in all areas [38]. As for our study, no relationship was found between the respondents’ marital status and their psychosocial status. Cancer affects both the patient’s physical state (through aggravating symptoms, such as pain, fatigue, and vomiting) and their mental state (mainly expressed through strong negative emotions). Cancer also necessitates changes in lifestyle and the social roles one fulfils. Being aware of your condition gives you a sense of control over your life and helps you adapt to a new and difficult situation. Mental adaptation to the disease is a process, the purpose of which is to alleviate emotional discomfort, and to regain mental stability. Therefore, adopting a proactive approach to the disease has an effect on the patient’s functioning in the physical, emotional, cognitive, and social dimensions [37,39]. As confirmed by numerous studies, patients’ negative perception of the disease negatively affects both their physical and mental states, reducing their quality of life [40,41,42,43]. However, de Rooj et al. observed that patients with endometrial cancer who suffered from more severe symptoms of the disease, and perceived the disease negatively due to survivorship care plans (SCPs), reported worse social and physical functioning, whereas patients with ovarian cancer who had a negative attitude towards the treatment process were characterized by worse emotional functioning [44]. It may be surprising, then, that, in our study, the patients who displayed weak acceptance of the disease reached higher scores for the physiological dimension compared to those who displayed moderate acceptance of the disease. Zielińska-Więczkowska et al. obtained different results, pointing out that, in oncological patients, difficulties with adapting and accepting the disease correlate with low quality of life in the physiological dimension [45].

Psychological care provided for patients diagnosed with gynaecological cancers will allow them to adopt constructive coping strategies, and manage the stress that accompanies every stage of the disease. Having the knowledge of the woman’s adaptation to cancer will help medical professionals make a diagnosis, evaluate her psychological, emotional, and social functioning, and intervene appropriately. All these elements are of key importance when ensuring and improving the quality of life of oncological patients.

## 5. Conclusions

1. The studied group of women diagnosed with gynaecological cancer displayed a moderate level of disease acceptance.
2. Constructive adaptive mechanisms were the most prevalent among the studied women.
3. The location of cancer had no bearing on the women’s adaptation to the disease and coping mechanisms.
4. Acceptance of the disease and an active attitude of the patients determined their adaptation to gynaecological cancer.

### Limitations of the Study

A limitation of the study is a relatively small number of participants. This is a result of the SARS-CoV2 pandemic and related sanitary-epidemiological restrictions, which reduced the assumed size of the study sample. However, this is a preliminary study. The authors see a need for further research in this area, and in the future plan to add additional aspects such as surgery, chemotherapy, and tumor progression.

## Figures and Tables

**Table 1:** Sociodemographic data with regard to the type of cancer.

Education (n, % *)					
Cancer Type	Higher	Primary	Secondary	Vocational	*p*
Ovarian Cancer	6 (26.1)	3 (13.0)	11 (47.8)	3 (13.0)	0.4631
Uterine Cancer	9 (15.5)	8 (13.8)	25 (43.1)	16 (27.6)	
Marital Status (n)					
Cancer Type	Divorced	Married	Single	Widowed	0.287
Ovarian Cancer	1 (4.3)	8 (34.8)	5 (21.7)	9 (39.10)	
Uterine Cancer	8 (13.8)	15 (25.9)	6 (10.3)	29 (50.0)	
Place of Residence (n)					
Cancer Type	City >100 K	City 10–100 K	City <10 K	Village	0.972
Ovarian Cancer	7 (30.4)	7 (30.4)	3 (13.0)	6 (26.1)	
Uterine Cancer	17 (29.3)	16 (27.6)	10 (17.2)	15 (25.9)	
Employment Status (n)					
Cancer Type	Pension	Unemployed	Retirement pension	Employed	0.419
Ovarian Cancer	1 (4.3)	2 (8.7)	9 (39.10)	11 (47.8)	
Uterine Cancer	3 (5.2)	3 (5.2)	34 (58.6)	18 (31.0)	

n—number, *p*—level of significance, * as per row total.

**Table 2:** Descriptive statistics for the Mini-Mac scale.

Variable	Whole Group (n = 81)	Ovarian Cancer (n = 23)	Uterine Cancer (n = 58)	*p*
	M ± SD	M ± SD	M ± SD	
Anxiety Preoccupation	18.26 ± 5.09	18.87 ± 5.54	18.02 ± 4.93	0.50
Helplessness/Hopelessness	13.31 ± 4.37	13.35 ± 5.22	13.29 ± 4.04	0.96
Fighting Spirit	22.99 ± 3.53	23.13 ± 3.32	22.93 ± 3.64	0.82
Positive Re-evaluation	22.12 ± 3.25	22.96 ± 2.64	21.79 ± 3.43	0.15
Constructive Style (PR + FS)	45.11 ± 6.01	46.09 ± 4.77	44.72 ± 6.43	0.36
Destructive Style (AP + HH)	31.57 ± 8.41	32.22 ± 9.59	31.31 ± 7.98	0.66

n—number, M—mean, SD—standard deviation, *p*—level of significance.

**Table 3:** Correlation between age and the scores on the Mini-Mac subscales.

Variable	Parameter	Whole Group (n = 81)	Ovarian Cancer (n = 23)	Uterine Cancer (n = 58)
Constructive Style	r	0.13	0.25	0.15
	*p*	0.24	0.24	0.28
Destructive Style	r	0.20	0.02	0.34
	*p*	0.07	0.94	0.01

*p*—level of significance; r—Pearson’s correlation.

**Table 4:** Constructive and destructive style categories depending on the type of cancer.

Variable	Constructive Style			*p*
Cancer Type (n, %)	High	Low	Moderate	
Ovarian Cancer	15 (65.2)	0 (0.0)	8 (23.8)	0.28
Uterine Cancer	29 (50.0)	4 (6.9)	25 (43.1)	
Destructive Style				
Cancer Type	High	Low	Moderate	
Ovarian Cancer	4 (17.4)	12 (52.2)	7 (30.4)	0.35
Uterine Cancer	5 (8.6)	27 (46.6)	26 (44.8)	

*p*—level of significance.

**Table 5:** Sociodemographic variables and level of disease acceptance.

Place of Residence	n (%)	M ± SD	*p*
City > 100 K	24 (29.6)	24.71 ± 9.35	0.54
City 10–100 K	23 (28.4)	27.43 ± 9.80	
City < 10 K	13 (16.0)	26.08 ± 9.47	
Village	21 (25.9)	28.38 ± 6.70	
Marital Status			
Divorced	9 (11.1)	29.67 ± 8.50	0.12
Married	23 (28.4)	29.57 ± 8.63	
Single	11 (13.6)	25.18 ± 9.23	
Widowed	38 (46.9)	24.61 ± 8.60	
Employment Status			
Pension	4 (4.9)	27.00 ± 6.78	0.87
Unemployed	5 (6.2)	24.60 ± 9.61	
Retirement pension	43 (53.1)	26.21 ± 9.18	
Employed	29 (35.8)	27.62 ± 8.79	
Education			
Higher	15 (18.5)	27.20 ± 7.67	0.881
Primary	11 (13.6)	27.00 ± 7.84	
Secondary	36 (44.4)	25.78 ± 9.90	
Vocational	19 (23.5)	27.68 ± 8.65	

n—number, M—mean, SD—standard deviation, *p*—level of significance.

**Table 6:** CAPS results and sociodemographic variables.

Variable	Physical Dimension			Self-Concept		Roles in Society	
Place of Residence	n	M ± SD	*p*	M ± SD	*p*	M ± SD	*p*
City > 100 K	24 (29.6)	36.58 ± 4.32	0.03	25.96 ± 2.27	0.17	18.79 ± 2.45	0.89
City 10–100 K	23 (28.4)	33.43 ± 5.67		26.13 ± 3.29		18.78 ± 3.07	
City < 10 K	13 (16.0)	38.85 ± 7.21		27.77 ± 3.19		19.38 ± 3.33	
Village	21 (25.9)	35.19 ± 4.43		25.38 ± 3.37		18.62 ± 2.80	
Marital Status							
Divorced	9 (11.1)	33.00 ± 7.18	0.27	27.22 ± 2.82	0.6	19.44 ± 2.24	0.66
Married	23 (28.4)	35.43 ± 4.17		26.43 ± 3.59		18.35 ± 2.67	
Single	11 (13.6)	34.73 ± 4.94		25.91 ± 2.59		18.45 ± 2.73	
Widowed	38 (46.9)	36.76 ± 5.85		25.79 ± 2.95		19.11 ± 3.10	
Employment Status							
Pension	4 (4.9)	33.75 ± 9.54	0.49	25.75 ± 4.03	0.89	17.50 ± 5.00	0.68
Unemployed	5 (6.2)	38.20 ± 2.17		25.20 ± 1.92		18.00 ± 1.87	
Retirement pension	43 (53.1)	36.14 ± 6.17		26.28 ± 3.22		19.02 ± 3.14	
Employed	29 (35.8)	34.86 ± 4.06		26.17 ± 2.99		18.90 ± 2.13	
Education							
Higher	15 (18.5)	33.93 ± 2.91	0.09	26.33 ± 2.72	0.86	19.53 ± 2.26	0.29
Primary	11 (13.6)	38.73 ± 5.87		26.64 ± 3.56		19.91 ± 1.81	
Secondary	36 (44.4)	34.89 ± 5.66		25.83 ± 2.40		18.31 ± 2.85	
Vocational	19 (23.5)	36.84 ± 5.99		26.32 ± 4.19		18.68 ± 3.51	
Variable	Coping			Interdependence		CAPS total	
Place of Residence							
City > 100 K	24 (29.6)	24.50 ± 2.50	0.36	30.63 ± 3.61	0.22	136.46 ± 9.71	0.24
City 10–100 K	23 (28.4)	24.87 ± 4.59		32.04 ± 4.60		135.26 ± 15.53	
City < 10 K	13 (16.0)	25.69 ± 4.55		32.08 ± 6.06		143.77 ± 21.46	
Village	21 (25.9)	23.48 ± 2.99		30.86 ± 3.93		133.52 ± 13.01	
Marital Status							
Divorced	9 (11.1)	25.33 ± 3.71	0.82	33.56 ± 4.33	0.27	138.56 ± 11.75	0.78
Married	23 (28.4)	24.00 ± 3.84		30.61 ± 4.53		134.83 ± 15.77	
Single	11 (13.6)	24.73 ± 3.69		30.09 ± 4.59		133.91 ± 15.04	
Widowed	38 (46.9)	24.61 ± 3.66		31.58 ± 4.23		137.84 ± 14.90	
Employment Status							
Pension	4 (4.9)	24.25 ± 5.68	0.35	30.25 ± 7.50	0.69	131.50 ± 20.04	0.89
Unemployed	5 (6.2)	24.20 ± 2.17		31.60 ± 3.78		137.20 ± 8.04	
Retirement pension	43 (53.1)	23.93 ± 3.99		30.88 ± 4.56		136.26 ± 16.91	
Employed	29 (35.8)	25.52 ± 2.98		32.07 ± 3.87		137.52 ± 11.47	
Education							
Higher	15 (18.5)	25.47 ± 2.70	0.62	32.27 ± 3.37	0.16	137.53 ± 10.20	0.24
Primary	11 (13.6)	24.64 ± 2.69		32.45 ± 3.08		142.36 ± 12.75	
Secondary	36 (44.4)	24.00 ± 4.05		30.08 ± 4.27		133.11 ± 14.38	
Vocational	19 (23.5)	24.74 ± 4.12		32.26 ± 5.57		138.84 ± 18.36	

n—number, M—mean, SD—standard deviation, *p*—level of significance.

**Table 7:** Correlations between the AIS and the CAPS scores.

Variable	Parameter	Whole Group (n = 81)	Ovarian Cancer (n = 23)	Uterine Cancer (n = 58)
CAPS Total Score	r	0.02	0.01	0.02
	*p*	0.84	0.95	0.91
Interdependence	r	0.15	0.09	0.16
	*p*	0.18	0.69	0.22
Physiological Dimension	r	−0.29	−0.26	−0.29
	*p*	0.01	0.23	0.03
Roles in Society	r	0.08	−0.19	0.14
	*p*	0.49	0.39	0.29
Self-concept	r	0.18	0.22	0.15
	*p*	0.11	0.31	0.27
Coping	r	0.13	0.21	0.08
	*p*	0.25	0.34	0.57

*p*—level of significance; r—Pearson’s correlation.

## Data Availability

The data presented in this study are available on request from the first author.
